# Joint Beam-Forming, User Clustering and Power Allocation for MIMO-NOMA Systems

**DOI:** 10.3390/s22031129

**Published:** 2022-02-02

**Authors:** Jiayin Wang, Yafeng Wang, Jiarun Yu

**Affiliations:** School of Information and Communication Engineering, Beijing University of Posts and Telecommunications, Beijing 100876, China; jiayinwang@bupt.edu.cn (J.W.); yujiarun@bupt.edu.cn (J.Y.)

**Keywords:** NOMA, MIMO, user clustering, power allocation, analog beam forming

## Abstract

In this paper, we consider the optimal resource allocation problem for multiple-input multiple-output non-orthogonal multiple access (MIMO-NOMA) systems, which consists of beam-forming, user clustering and power allocation, respectively. Users can be divided into different clusters, and the users in the same cluster are served by the same beam vector. Inter-cluster orthogonality can be guaranteed based on multi-user detection (MUD). In this paper, we propose a three-step framework to solve the multi-dimensional resource allocation problem. In step 1, we propose a beam-forming algorithm for a given user cluster. Specifically, fractional transmitting power control (FTPC) is applied for intra-cluster power allocation. The considered beam-forming problem can be transformed into a non-constrained one and the limited-memory Broyden–Fletcher–Goldfarb–Shanno (L-BFGS) method is applied to obtain the optimal solution. In step 2, optimal user clustering is further considered. Channel differences and correlations are both involved in the design of user clustering. By assigning different weights to the two factors, we can produce multiple candidate clustering schemes. Based on the proposed beam-forming algorithm, beam-forming can be done for each candidate clustering scheme to compare their performances. Moreover, based on the optimal user clustering and beam-forming schemes, in step 3, power allocation can be further optimized. Specifically, it can be formalized as a difference of convex (DC) programming problem, which is solved by successive convex approximation (SCA) with strong robustness. Simulations results show that the proposed scheme can effectively improve spectral efficiency (SE) and edge users’ data rates.

## 1. Introduction

Traditional orthogonal multiple access (OMA) has met a bottleneck, since the limited spectrum resources cannot meet the ever-growing demand for mobile data traffic. As an alternative, non-orthogonal multiple access (NOMA) has attracted considerable attention since it allows multiple users to occupy the same spectrum resource simultaneously. According to NOMA protocols, users can be divided into different clusters based on their channel characteristics. The signals of the users in the same cluster will be further transmitted utilizing the same time-frequency resource [1]. In each cluster, the channel differences among different users should be large enough to perform successive interference cancellation (SIC) successfully [2,3,4]. Moreover, weak users can be compensated in the power allocation process, which not only improves edge users’ performances, but helps to better identify multiplexed users in the power domain [5,6,7,8].

Moreover, multiple-input multiple-output (MIMO) also serves as a promising technique by which to multiply the spectrum efficiency (SE) gain [9,10,11]. In massive MIMO systems, beam-forming can effectively improve SE based on spacial diversity [12]. Conventionally, a specific beam vector can be designed for each user. The interference among multiple users can be eliminated when the number of antennas is greater than that of users. Specifically, the beam vector of each user can be set orthogonal to the channel vectors of others based on the zero-forcing beam-forming (ZF-BF) algorithm [13,14].

In this passage, SE can be further improved by exploiting both the spacial and power domain, i.e., MIMO-NOMA. We divide users into different clusters and further design a beam vector for each cluster. For each user, the inter-cluster interference can be transformed into the inter-beam interference, which is further eliminated based on ZF-BF [14,15,16]. Moreover, the beam vector of each cluster mainly depends on the channel characteristics of strong intra-cluster users [17]. The gaps between strong users and weak users will continue to widen, which is favored by NOMA.

### 1.1. Related Works

MIMO-NOMA has received considerable research interest for its ability to improve SE. Optimal user clustering for a downlink NOMA system was considered in [4], where the users were divided into different clusters based on an improved sorting algorithm. The authors of [5] applied NOMA to a MIMO system and demonstrated that the combined application can bring extra SE improvements only when the channel correlations among multiplexed users were sufficiently high. In [9], beam-forming and power allocation were jointly optimized for MIMO-NOMA based on semi-definite programming (SDP). In [12], simultaneous wireless information and power transfer (SWIPT) was applied in cooperation with NOMA, with the aim of enhancing edge users’ data rates. The angle domain was exploited in [16] to identify those users occupying the same spectrum resources, and the beam-forming problem was further considered based on an estimation of users’ angle information. In [17], receiver antenna selection (RAS) was applied in an uplink MIMO-NOMA system to ensure that cell-edge users could be more likely to participate in the communication process. The authors of [18] proposed a beam-forming algorithm for MIMO-NOMA. An effective channel vector was obtained for each cluster to describe the channel characteristics of intra-cluster users, which provided a compatible dimension for ZF-BF. Moreover, high-speed beam-forming for MIMO-NOMA was studied in [19]. The authors of [20] integrated device-to-device (D2D) communications with MIMO-NOMA to further improve SE. Consequently, in [20], a novel resource allocation scheme was proposed for the integrated system to overcome interference. Research on the problem of resource allocation in NOMA has also been expanded to a multi-cell scenario. [21] investigated the resource allocation problem for multi-cell MIMO-NOMA-based internet of things (IoT) networks. Moreover, [22] investigated the energy efficiency (EE) maximization problem for multi-cell, massive MIMO-NOMA networks with wireless power transfer. The authors of [22] proposed a novel joint power, time, antenna and subcarrier allocation scheme, which could properly allocate the time for energy harvesting and data transmission.

### 1.2. Our Contributions

In MIMO-NOMA systems, SIC is of great significance in reducing intra-cluster interference. While a user is decoding the signals of others based on SIC, past research has tended to set a lower bound for the received signal to interference and noise ratio (SINR) to ensure the decoding process goes smoothly. In [23], the optimal power allocation scheme for the downlink of NOMA system was obtained based on Karush-Kuhn-Tucker (KKT) conditions with a SINR bound of 0.3. Additionally, [9] jointly optimized power allocation and beam-forming for MIMO-NOMA with a SINR bound less than 0.5. Unfortunately, most related works fail to obtain a feasible solution when the SINR bound is greater than 1, which makes the received SINR for users relatively lower and, in turn, decreases system reliability. One explanation for this is that, most related works usually consider the joint optimization of power allocation and beam-forming. The scale of the considered problem is relatively large, which makes it challenging for optimization tools to obtain a feasible solution. To address this issue, we decompose the multi-dimensional resource allocation problem into three sub-problems. The scope of each sub-problem is relatively small, which helps to obtain a feasible solution with strong robustness.

Moreover, most existing works only consider channel difference characteristics while determining the clustering scheme for MIMO-NOMA. However, since the users in the same cluster are served by the same beam vector, their channel correlations should be relatively high to bring the advantages of MIMO into full play. The clustering criterion in [9,10,11,12] was to make the channel differences among multiplexed users as big as possible, which neglected channel correlations characteristics and was not directly related to the ultimate system performance. In this paper, channel correlations and differences are both involved in the design of user clustering. By assigning different weights to the two factors, we can produce multiple possible clustering schemes. Beam-forming can be done for each possible clustering scheme to compare their performance, which ensures the ultimate clustering scheme achieves the maximum SE performance.

In addition, some related literature only considers the resource allocation problem for a two-user-cluster. In this paper, the size of each cluster is not fixed, which makes the proposed scheme more practical. The main contributions of this paper are summarized as below:

1. We present a system model for MIMO-NOMA. Multiple users can be divided into different clusters and the size of each cluster is not fixed. The users in the same cluster are served by the same beam vector. Each user is assumed to detect signals based on a specific receiving coefficient to ensure inter-cluster orthogonality. Moreover, SIC is applied to users to alleviate intra-cluster interference.
2. We propose a three-step framework to solve the multi-dimensional resource allocation problem. In step 1, a beam-forming algorithm is proposed to obtain the optimal beam vector for a given user cluster. Specifically, fractional transmitting power control (FTPC) is applied to perform intra-cluster power allocation. The considered beam-forming problem can be transformed into a non-constrained one and the limited-memory Broyden–Fletcher–Goldfarb–Shanno (L-BFGS) method is applied to obtain a local optimal with less complexity.
3. In step 2, user clustering is further considered, based on the proposed beam-forming algorithm. For each user *k*, we define a utility function to describe its preference on each cluster *n*. The utility function consists of two terms, which depict the channel differences and correlations between user *k* and the existing users in cluster *n*, respectively. A relative weight is introduced for the two factors to balance the tradeoff between channel differences and correlations. Based on the utility function, user *k* can be further assigned to its favorite cluster. In this paper, the relative weight is obtained by particle swarm optimization (PSO). In PSO, we can simultaneously produce multiple possible solutions for the relative weight, each corresponding to a possible clustering scheme. Based on the proposed beam-forming algorithm, beam-forming can be done for each possible clustering scheme to compare their performance, which ensures the ultimate clustering scheme achieves the maximum SE performance.
4. In step 3, power allocation is further optimized based on the optimal user clustering and beam-forming schemes. As mentioned before, it can be formalized as a difference of convex (DC) programming problem utilizing the specific characteristic of the objective function, which can be solved by successive convex approximation (SCA) through limited iterations. We evaluate the performance of the proposed scheme and some other existing schemes to illustrate the significance of the proposed scheme.

The rest of the paper is organized as follows: Section 2 presents the system model for MIMO-NOMA and further provides a mathematical expression of the optimal resource allocation problem. Section 3 introduces more details about the proposed beam-forming algorithm. Section 4 and Section 5 introduce the user clustering and power allocation schemes, respectively. The performance of the proposed scheme is evaluated in Section 6. Section 7 concludes this paper.

## 2. System Model

Consider a single-cell downlink MIMO-NOMA system, in which there is one base station (BS) equipped with *N* antennas and *K* single-antenna users. Let U={1,2,…,K} denote the set of users. Without loss of generality, the users are indexed by the descending order of channel gains, i.e., ${\left|h_{1}\right|}^{2}>{\left|h_{2}\right|}^{2}>\ldots >{\left|h_{K}\right|}^{2}$, where $h_{k}\in C^{N\times 1}$ (k∈{1,2,…,K}) denotes the channel vector of user *k*. All the *K* users will be further divided into *S* different clusters. Let $U_{n}=\left\{i_{n}\left(1\right),i_{n}\left(2\right),\ldots ,i_{n}\left(m_{n}\right)\right\}$ (n∈{1,2,…,S}) denote the set of users assigned to cluster *n*, where $m_{n}$ denotes the size of $U_{n}$, and $i_{n}\left(l\right)$ ($l\in \{1,2,\ldots ,m_{n}\}$) denotes the index of the *l*-th user in $U_{n}$. Specifically, the users in $U_{n}$ are sorted in the ascending order of their indexes, i.e., $i_{n}\left(1\right)<i_{n}\left(2\right)<\ldots <i_{n}\left(m_{n}\right)$. The superposed signal at the BS is given by

(1) $$\mathrm{ss}=\sum_{n=1}^{S}w_{n}\sum_{l=1}^{m_{n}}\sqrt{p_{i_{n}\left(l\right)}}s_{i_{n}\left(l\right)}$$

where $w_{n}\in C^{N\times 1}$ denotes the beam vector of cluster *n*, $s_{i_{n}\left(l\right)}$ and $p_{i_{n}\left(l\right)}$ denote the signal and power of user $i_{n}\left(l\right)$, respectively. Assume the beam vector of each cluster has constant modulus (CM) elements. The received signal at user $i_{n}\left(l\right)$ is given by

(2) $$\begin{array}{cc} r_{i_{n}\left(l\right)}= & {h_{i_{n}\left(l\right)}}^{H}w_{n}\sqrt{p_{i_{n}\left(l\right)}}s_{i_{n}\left(l\right)}\\ \; & +\underset{\mathrm{inter}-\mathrm{cluster}-\mathrm{interference}}{\underset{︸}{{h_{i_{n}\left(l\right)}}^{H}\sum_{q\in \{1,2,\ldots ,S\}\backslash \{n\}}w_{q}\sum_{a=1}^{m_{q}}\sqrt{p_{i_{q}\left(a\right)}}s_{i_{q}\left(a\right)}}}\\ \; & +\underset{\mathrm{intra}-\mathrm{cluster}-\mathrm{interference}}{\underset{︸}{{h_{i_{n}\left(l\right)}}^{H}w_{n}\sum_{b\in \left\{1,2,\ldots ,m_{n}\right\}\backslash \left\{l\right\}}\sqrt{p_{i_{n}\left(b\right)}}s_{i_{n}\left(b\right)}}}+\omega_{i_{n}\left(l\right)} \end{array}$$

where $h_{i_{n}\left(l\right)}$ denotes the channel vector of user $i_{n}\left(l\right)$. The first term in (2) represents the received desired signal. The second and third term represent the inter-cluster and intra-cluster interference, respectively. The noise term $\omega_{i_{n}\left(l\right)}$ is a zero-mean complex additive white Gaussian noise (AWGN) with variance $\sigma^{2}$. One can observe that the received interference is significantly larger. To solve this problem, each user is assumed to detect signals via a specific receiving coefficient, given by

(3) $$\alpha_{i_{n}\left(l\right)}=v_{i_{n}\left(l\right)}^{H}h_{i_{n}\left(l\right)}$$

where $\alpha_{i_{n}\left(l\right)}$ denotes the receiving coefficient of user $i_{n}\left(l\right)$, $v_{i_{n}\left(l\right)}\in C^{N\times 1}$. Then, the received signal at user $i_{n}\left(l\right)$ can be re-written as

(4) $$\begin{array}{cc} {\bar{r}}_{i_{n}\left(l\right)}= & \alpha_{i_{n}\left(l\right)}r_{i_{n}\left(l\right)}\\ = & v_{i_{n}\left(l\right)}^{H}H_{i_{n}\left(l\right)}w_{n}\sqrt{p_{i_{n}\left(l\right)}}s_{i_{n}\left(l\right)}\\ \; & +v_{i_{n}\left(l\right)}^{H}H_{i_{n}\left(l\right)}\sum_{q\in \{1,2,\ldots ,S\}\backslash \{n\}}w_{q}\sum_{a=1}^{m_{q}}\sqrt{p_{i_{q}\left(a\right)}}s_{i_{q}\left(a\right)}\\ \; & +v_{i_{n}\left(l\right)}^{H}H_{i_{n}\left(l\right)}w_{n}\sum_{b\in \left\{1,2,\ldots ,m_{n}\right\}\backslash \left\{l\right\}}\sqrt{p_{i_{n}\left(b\right)}}s_{i_{n}\left(b\right)}+{\bar{\omega }}_{i_{n}\left(l\right)} \end{array}$$

where $H_{i_{n}\left(l\right)}=h_{i_{n}\left(l\right)}h_{i_{n}\left(l\right)}^{H}$. For user $i_{n}\left(l\right)$, the interference from cluster *q* (q≠n) can be eliminated when the following condition satisfies:

(5) $$v_{i_{n}\left(l\right)}^{H}H_{i_{n}\left(l\right)}w_{q}=0$$

Let ${\tilde{h}}_{n,l}^{q}=H_{i_{n}\left(l\right)}w_{q}$, and let ${\tilde{H}}_{n,l}=\left[{\tilde{h}}_{n,l}^{1},{\tilde{h}}_{n,l}^{2},\ldots ,{\tilde{h}}_{n,l}^{n-1},{\tilde{h}}_{n,l}^{n+1},\ldots ,{\tilde{h}}_{n,l}^{S}\right]$. For user $i_{n}\left(l\right)$, the inter-cluster interference can be totally eliminated by setting $v_{i_{n}\left(l\right)}$ as the left singular vector of ${\tilde{H}}_{n,l}$ corresponding to the zero singular value. It is worth noting that there is a constraint for this operation, i.e., N≥S−1. In addition, $\alpha_{i_{n}\left(l\right)}$ should be normalized to ensure that it will not bring extra SE gains, i.e., $\left|v_{i_{n}\left(l\right)}^{H}h_{i_{n}\left(l\right)}\right|=1$.

Accordingly, the received signal at user $i_{n}\left(l\right)$ can be transformed into

(6) $$\begin{array}{cc} {\bar{r}}_{i_{n}\left(l\right)}= & v_{i_{n}\left(l\right)}^{H}H_{i_{n}\left(l\right)}w_{n}\sqrt{p_{i_{n}\left(l\right)}}s_{i_{n}\left(l\right)}\\ \; & +v_{i_{n}\left(l\right)}^{H}H_{i_{n}\left(l\right)}w_{n}\sum_{b\in \left\{1,2,\ldots ,m_{n}\right\}\backslash \left\{l\right\}}\sqrt{p_{i_{n}\left(b\right)}}s_{i_{n}\left(b\right)}+{\bar{\omega }}_{i_{n}\left(l\right)} \end{array}$$

Moreover, SIC is performed at users to further reduce intra-cluster interference. According to SIC, in each cluster, a user can decode the signals of the others with poorer channel conditions. Conventionally, since $U_{n}$ is a sorted sequence, user $i_{n}\left(b\right)$ ($b\in \left\{1,2,\ldots ,m_{n}\right\}\backslash \left\{l\right\}$) can decode the signals of user $i_{n}\left(l\right)$ if and only if *b* < *l*. However, in MIMO-NOMA system, users’ channel gains depend not only on the physical environments but on the beams, i.e., the decoding priority may not be fixed and is subject to the beam-forming scheme. Specifically, user $i_{n}\left(b\right)$ can decode the signals of user $i_{n}\left(l\right)$ when the following condition satisfies:

(7) $$w_{n}^{H}h_{i_{n}\left(b\right)}h_{i_{n}\left(b\right)}^{H}w_{n}-w_{n}^{H}h_{i_{n}\left(l\right)}h_{i_{n}\left(l\right)}^{H}w_{n}>0$$

Obviously, beam-forming affects the decoding order by adjusting users’ effective channel gains. Accordingly, we introduce a decoding indicator $\lambda_{n}^{b,l}$ to depict whether or not user $i_{n}\left(b\right)$ can decode the signals of user $i_{n}\left(l\right)$, given by

(8) $$\lambda_{n}^{b,l}=\frac{1}{2}\left(1+\mathrm{sgn}\left(w_{n}^{H}h_{i_{n}\left(b\right)}h_{i_{n}\left(b\right)}^{H}w_{n}-w_{n}^{H}h_{i_{n}\left(l\right)}h_{i_{n}\left(l\right)}^{H}w_{n}\right)\right)$$

Here, the sign function is introduced to denote the decoding priority, which returns 1 when its input is positive, and −1 otherwise. From (8), if user $i_{n}\left(b\right)$ can decode the signals of user $i_{n}\left(l\right)$, $\lambda_{n}^{b,l}=1$; otherwise, $\lambda_{n}^{b,l}=0$. When $\lambda_{n}^{b,l}=1$, there is an implicit power constraint, given by

(9) $$p_{i_{n}\left(l\right)}-p_{i_{n}\left(b\right)}>0$$

From (9), when $\lambda_{n}^{b,l}=1$, the power of user $i_{n}\left(l\right)$ should be larger than that of user $i_{n}\left(b\right)$ to make $i_{n}\left(l\right)$ more easily detected. The received SINR at user $i_{n}\left(l\right)$ can be expressed as

(10) $$SINR_{i_{n}\left(l\right)}=\frac{p_{i_{n}\left(l\right)}g_{i_{n}\left(l\right)}}{1+\sum_{b\in \left\{1,2,\ldots ,m_{n}\right\}\backslash \left\{l\right\}}\lambda_{n}^{b,l}p_{i_{n}\left(b\right)}g_{i_{n}\left(l\right)}}$$

where $g_{i_{n}\left(l\right)}=\frac{w_{n}^{H}h_{i_{n}\left(l\right)}h_{i_{n}\left(l\right)}^{H}w_{n}}{{\left|{\bar{\omega }}_{i_{n}\left(l\right)}\right|}^{2}}$ denotes the normalized channel gain of user $i_{n}\left(l\right)$. Based on the discussion above, the considered problem can be mathematically expressed as below:

(11a) $$\max_{P,W,I}:\sum_{n=1}^{S}\sum_{l=1}^{m_{n}}\log(1+\frac{p_{i_{n}\left(l\right)}g_{i_{n}\left(l\right)}}{1+\sum_{b\in \left\{1,2,\ldots ,m_{n}\right\}\backslash \left\{l\right\}}\lambda_{n}^{b,l}p_{i_{n}\left(b\right)}g_{i_{n}\left(l\right)}})$$

(11b) $$s.t.\frac{p_{i_{n}\left(l\right)}g_{i_{n}\left(b\right)}}{1+\sum_{j\in \left\{1,2,\ldots ,m_{n}\right\}\backslash \left\{l\right\}}\lambda_{n}^{j,l}p_{i_{n}\left(j\right)}g_{i_{n}\left(b\right)}}>\Gamma ,\lambda_{n}^{b,l}=1$$

(11c) $$p_{i_{n}\left(l\right)}-p_{i_{n}\left(b\right)}>0,\lambda_{n}^{b,l}=1$$

(11d) $$\left|{\left[w_{n}\right]}_{c}\right|=\frac{1}{\sqrt{N}},\forall n,\forall c=1,2,\ldots ,N$$

(11e) $$\sum_{n=1}^{S}\sum_{l=1}^{m_{n}}p_{i_{n}\left(l\right)}\leq P_{tot}$$

(11f) $$m_{n}\leq M,\forall n$$

where $P=\{p_{k},k=1,2,\ldots ,K\}$, $W=\{w_{n},\forall n\}$ and $I=\{U_{n},\forall n\}$ denote the power allocation, beam-forming and user clustering schemes for MIMO-NOMA, respectively. (11b) denotes the constraint on SIC, where Γ denotes the SINR threshold for a successful decoding. (11c) denotes the implicit power constraint. (11d) denotes the CM constraint, where ${\left[w_{n}\right]}_{c}$ denotes the *c*-th element in $w_{n}$. Constraint (11e) provides power budget $P_{tot}$ for the considered system. Constraint (11f) indicates that each NOMA cluster can serve at most *M* users.

## 3. Beam-Forming Algorithm for a Given User Cluster

Problem (11) considers the joint optimization of user clustering, beam-forming and power allocation for MIMO-NOMA, which is challenging to be solved in a polynomial time. Due to the orthogonality among different clusters, in this section, we first consider the beam-forming problem for a given user cluster (the optimal user clustering and power allocation schemes will be further discussed in Section 4 and Section 5, respectively). Without loss of generality, we assign the first *m* users of U to cluster *n*, i.e., $i_{n}\left(l\right)=l,\forall l=1,2,\ldots ,m$. The beam-forming problem for *n* can be mathematically expressed as below:

(12a) $$\max_{w_{n},P_{n}}:\sum_{l=1}^{m}\log(1+\frac{p_{l}g_{l}}{1+\sum_{b=\{1,2,\ldots ,m\}\backslash \{l\}}\lambda_{n}^{b,l}p_{b}g_{l}})$$

(12b) $$s.t.\lambda_{n}^{b,l}=\frac{1}{2}\left(1+\mathrm{sgn}\left(w_{n}^{H}h_{b}h_{b}^{H}w_{n}-w_{n}^{H}h_{l}h_{l}^{H}w_{n}\right)\right),\forall b,l\in \left\{1,2,\ldots ,m\right\},b\neq l$$

(12c) $$p_{l}-p_{b}>0,\lambda_{n}^{b,l}=1$$

(12d) $$\sum_{l=1}^{m}p_{l}\leq \frac{P_{tot}}{S}$$

(12e) $$\left|{\left[w_{n}\right]}_{c}\right|=\frac{1}{\sqrt{N}},\forall c=1,2,\ldots ,N$$

where $P_{n}=\left\{p_{l},\forall l=1,2,\ldots ,m\right\}$ denotes the power allocation scheme for the *m* considered users. For the sake of simplify, the SINR constraint is omitted here and will be further considered in Section 5. Each cluster is assumed to have the same power budget, denoted by $\frac{P_{tot}}{S}$. Due to (12e), the beam vector can be represented as $w_{n}=\frac{1}{\sqrt{N}}{\left(e^{j\phi_{1}},e^{j\phi_{2}},\ldots ,e^{j\phi_{N}}\right)}^{T}$, where $\phi_{c}$ denotes the phase of the *c*-th element in $w_{n}$. The beam vector is obtained once the phases of its elements are determined. Inspired by this observation, we treat $\Phi =[\phi_{1},\phi_{2},\ldots ,\phi_{N}]$ as variables. Based on perfect square formula, users’ normalized channel gains can be expressed in terms of Φ (for the details of derivation, see Appendix A).

(13) $$g_{l}=\frac{1}{{\left|{\bar{\omega }}_{l}\right|}^{2}N}{∥h_{l}∥}_{2}^{2}+\frac{2}{{\left|{\bar{\omega }}_{l}\right|}^{2}N}\sum_{c=1}^{N}\sum_{d=c+1}^{N}\kappa_{l,c}\kappa_{l,d}\cos(\phi_{c}-\phi_{d}-\left(\varphi_{l,c}-\varphi_{l,d}\right)$$

where $\kappa_{l,c}$ and $\varphi_{l,c}$ denote the amplitude and phase of the *c*-th element in $h_{l}$, respectively. However, problem (12) is still difficult to solve due to (12c) and (12d). To predigest the scope of (12), we first produce a feasible solution for $P_{n}$ and then maximize (12a) by optimizing Φ. Specifically, the power allocation scheme can be obtained based on FTPC, i.e., the transmit power of user *l* can be represented by:

(14) $$p_{l}=\frac{P_{tot}}{S}\frac{g_{l}^{-\gamma }}{\sum_{j=1}^{m}g_{j}^{-\gamma }}$$

where γ denotes the decay factor. With FTPC, constraint (12d) always holds since $\sum_{l}p_{l}=\frac{P_{tot}}{S}$. Moreover, γ determines the correlation between users’ channel gains and transmitting power. When γ=0, transmitting power is totally unrelated to normalized gains, i.e., each user has the same transmitting power. Moreover, $p_{l}$ and $g_{l}$ will be negatively correlated as γ increases, which is consistent with (12c) and thus makes (14) a feasible solution.

Accordingly, problem (12) can be transformed into

(15) $$\max_{\Phi }:\sum_{l=1}^{m}\log(1+\frac{\frac{P_{tot}}{S}g_{l}^{1-\gamma }}{\sum_{j=1}^{m}g_{j}^{-\gamma }+\frac{P_{tot}}{S}\sum_{b\in \{1,2,\ldots ,m\}\backslash \{l\}}\lambda_{n}^{b,l}g_{b}^{-\gamma }g_{l}})$$

However, it is still challenging for us to solve (15) since the sign function in (12b) is non-differentiable. To solve this problem, we produce an approximation of $\lambda_{n}^{b,l}$, given by

(16) $${\bar{\lambda }}_{n}^{b,l}=\frac{1}{1+\exp(g_{l}-g_{b})}$$

The Sigmoid function is introduced which is first-order differentiable. From (16), when $g_{b}-g_{l}\to +\infty$, ${\bar{\lambda }}_{n}^{b,l}\to 1$; when $g_{b}-g_{l}\to -\infty$, ${\bar{\lambda }}_{n}^{b,l}\to 0$. Since the output of (16) ranges from zero to one, we consider (16) as the probability that user *b* successfully decodes the signals of user *l*.

Then, (15) can be re-written as

(17) $$\max_{\Phi }:f\left(\Phi \right)=\sum_{l=1}^{m}\log(1+\frac{\frac{P_{tot}}{S}g_{l}^{1-\gamma }}{\sum_{j=1}^{m}g_{j}^{-\gamma }+\frac{P_{tot}}{S}\sum_{b\in \{1,2,\ldots ,m\}\backslash \{l\}}\frac{g_{b}^{-\gamma }g_{l}}{1+\exp(g_{l}-g_{b})}})$$

Consider the partial derivatives:

(18) $$\frac{\partial f}{\partial \phi_{c}}=\sum_{l=1}^{m}\frac{\partial f}{\partial g_{l}}\frac{\partial g_{l}}{\partial \phi_{c}}$$

(19) $$\frac{\partial g_{l}}{\partial \phi_{c}}=\frac{2}{{\bar{\omega }}_{l}N}\sum_{d=1}^{N}\kappa_{l,d}\kappa_{l,c}\sin\left(\phi_{d}-\varphi_{l,d}-\left(\phi_{c}-\varphi_{l,c}\right)\right)$$

Since problem (17) is a differentiable non-constrained problem, a quasi-Newton method named L-BFGS can be applied to solve it in limited iterations. In each iteration, L-BFGS produces an updating direction for Φ based on the information from the last *T* iterations. Once the update direction is determined, the Armijo rule is applied to obtain a proper step size. More details about the proposed algorithm are as shown in Algorithm 1.
**Algorithm 1** Beam-forming Algorithm for A Given Cluster**Require:** 
$U_{n}$**Ensure:** 
Φ1:Initialize *T*, η.2:$Y_{n}$ = Ø, $S_{n}$ = Ø, $R_{n}$ = Ø.3:Randomly initialize Φ.4:$g_{\mathrm{pre}}$← the gradient of *f* at Φ.5:Calculate the updating direction y= −$g_{\mathrm{pre}}$.6:Obtain the optimal step size μ based on the Armijo rule.7:y←μy, Φ←Φ+y.8:$g_{\mathrm{cur}}$← the gradient of *f* at Φ.9:$s\leftarrow g_{\mathrm{cur}}-g_{\mathrm{pre}}$.10:$\rho \leftarrow y^{H}s$.11:**while** $\left|g_{\mathrm{cur}}\right|\geq \eta$ **do**12:   $g_{\mathrm{pre}}\leftarrow g_{\mathrm{cur}}$.13:   Insert y to $Y_{n}$.14:   Insert s to $S_{n}$.15:   Insert ρ to $R_{n}$.16:   *L*← the number of the elements in $Y_{n}$.17:   **if** L>T **then**18:     Pop the first element in $Y_{n}$.19:     Pop the first element in $S_{n}$.20:     Pop the first element in $R_{n}$.21:     *L*←L−1.22:   **end if**23:   (Back Propagating)24:   **for** *i* = *L*:−1:1 **do**25:     s← the *i*-th element in $S_{n}$.26:     y← the *i*-th element in $Y_{n}$.27:     ρ← the *i*-th element in $R_{n}$.28:     $\chi_{i}\leftarrow \rho s^{H}g_{\mathrm{cur}}$.29:     $g_{\mathrm{cur}}\leftarrow g_{\mathrm{cur}}-\chi_{i}y$.30:   **end for**31:   (Forward Propagating)32:   $\mathrm{res}\leftarrow g_{\mathrm{cur}}$.33:   **for** *i* = 1:*L* **do**34:     s← the *i*-th element in $S_{n}$.35:     y← the *i*-th element in $Y_{n}$.36:     ρ← the *i*-th element in $R_{n}$.37:     $\beta_{i}\leftarrow \rho y^{H}\mathrm{res}$.38:     $\mathrm{res}\leftarrow \mathrm{res}+(\chi_{i}-\beta_{i})s$.39:   **end for**40:   y=−res.41:   steps (6)–(10)42:**end while**

## 4. User Clustering for MIMO-NOMA System

In this section, optimal user clustering is further considered based on Algorithm 1. In each cluster, the channel differences among multiplexed users should be large enough to perform SIC successfully. Moreover, since the users in the same cluster are served by the same beam vector, their channel correlations should also be emphasized to bring the advantages of MIMO into full play. Accordingly, the optimal clustering scheme will be obtained with consideration for both the two factors.

Due to SIC, the strongest user in each cluster is in fact served by OMA, which can achieve good performance with less power when its channel gain is relatively large. Therefore, the first *S* users of U will be assigned to *S* different clusters, respectively. Due to the high channel gains, these users could achieve good performances with less power, which can in turn enable more power budget for others. After initializing *S* clusters, the remaining users in U will successively select a suitable cluster to join. For each user *k*, we define a utility function to assess its preference on different clusters, given by

(20) $$u_{k}\left(n\right)=\frac{1}{m_{n}}\sum_{l=1}^{m_{n}}\frac{\left|h_{k}^{H}h_{i_{n}\left(l\right)}\right|}{\left|h_{k}\right|\left|h_{i_{n}\left(l\right)}\right|}-\theta \frac{{\left(\left|h_{k}\right|+\sum_{l=1}^{m_{n}}\;\left|h_{i_{n}\left(l\right)}\right|\right)}^{2}}{\left(m_{n}+1\right)\left({\left|h_{k}\right|}^{2}+\sum_{l=1}^{m_{n}}{\left|h_{i_{n}\left(l\right)}\right|}^{2}\right)}$$

where $u_{k}\left(n\right)$ describes user *k*’s preference for cluster *n*. The first term depicts the channel correlations between user *k* and the existing users in cluster *n*. The second term is the Jain’s fairness index, which measures the channel difference between user *k* and the existing users in cluster *n*. Specifically, the second term ranges from $\frac{1}{m_{n}+1}$ to 1 and will decrease as the channel difference gets larger. θ denotes the relative weight for the two aspects. Based on the utility function, user *k* will be further assigned to its favorite cluster $n_{k}$, given by

(21) $$n_{k}=\underset{n\in \{1,2,\ldots ,S\}}{\arg\max}\left(u_{k}\left(n\right)\right)$$

Clusters will reject users only when condition (11f) is not met. For each cluster *n*, when $m_{n}=M+1$, *n* should reject a user to satisfy the size-constraint. Accordingly, we can produce multiple possible user set for cluster *n* by removing any single user from $U_{n}$. Based on Algorithm 1, beam-forming can be done for each possible user set to compare their performances, and the optimal user set for cluster *n* is obtained accordingly.

One can observe that the relative weight is of great significance in steering the ultimate clustering scheme. When θ is relatively small, channel correlations play a decisive role in the clustering process. As θ increases, channel differences, in turn, become the controlling factor of the ultimate clustering scheme. With any given θ, user clustering can be performed based on Algorithm 2. Then, Algorithm 1 can be applied to obtain a beam-forming scheme, and the corresponding achievable SE can be denoted by w(θ).

In this section, the optimal θ is obtained by PSO. In PSO, the optimal θ can be obtained through numerous iterations. In each iteration, PSO produces multiple possible solutions for θ, each corresponding to a possible clustering scheme. Based on Algorithm 1, beam-forming can be done for each possible clustering scheme to compare their performance. We will further select the one with the maximum SE performance as the optimal clustering scheme, and its corresponding relative weight is exactly the optimal θ obtained by PSO. More details are as described in Algorithm 3. Note that the random variable δ in step (13) denotes the step size, which is a real number ranging from 0 to 1.
**Algorithm 2** User Clustering Scheme for MIMO-NOMA with A Given Relative Weight1:Assign the first *S* users of U to *S* different clusters.2:Construct the utility function as (20) based on the given relative weight.3:**for***j*=*S* + 1:*K* **do**4:   Sort multiple clusters based on user *j*’s preference.5:   Denote the sorted sequence by $\Omega_{j}$.6:   **while** $\Omega_{j}$ ≠Ø **do**7:     $n_{j}$← the first cluster in $\Omega_{j}$.8:     Insert user *j* to $U_{n_{j}}$.9:     NUM← the number of the users in $U_{n_{j}}$.10:     **if** NUM≤M **then**11:        Break.12:     **else**13:        **for** *i* = 1:*M* + 1 **do**14:          Remove the *i*-th user from $U_{n_{j}}$.15:          Obtain the optimal beam vector for cluster $n_{j}$ by Algorithm 1.16:          $\varepsilon_{i}$← the sum rate of the users in $U_{n_{j}}$.17:          Insert the removed user to its original position.18:        **end for**19:        $\tilde{i}\leftarrow$ the position of the maximum in $\left[\varepsilon_{1},\varepsilon_{2},\ldots ,\varepsilon_{M+1}\right]$.20:        x← the index of the $\tilde{i}$-th user in $U_{n_{j}}$.21:        Remove the $\tilde{i}$-th user from $U_{n_{j}}$.22:     **end if**23:     **if** x=j **then**24:        Remove cluster $n_{j}$ from $\Omega_{j}$.25:     **else**26:        Break.27:     **end if**28:   **end while**29:**end for**

**Algorithm 3** PSO-based Optimal User Clustering
1:Initialize group size *G*.2:Initialize the total number of iterations *D*.3:**for** *g* = 1:*G* **do**4:    Initialize position $\theta_{g}$ for particle *g*.5:    $e_{best,g}=w\left(\theta_{g}\right)$.6:    $p_{best,g}=\theta_{g}$.7:
**end for**
8:$\bar{g}\leftarrow$ the position of the maximum in $\left\{e_{best,g},\forall g\right\}$.9:$G_{best}=p_{best,\bar{g}}$.10:*t* = 1.11:**while** t≤D **do**12:   **for** *g* = 1:*G* **do**13:      $\theta_{g}=0.5\left(p_{best,g}+G_{best}\right)+\delta \left(p_{best,g}-G_{best}\right)$.14:      $e_{g}=w\left(\theta_{g}\right)$.15:      **if** $e_{g}>e_{best,g}$ **then**16:         $p_{best,g}=\theta_{g}$.17:         $e_{best,g}=e_{g}$.18:      **end if**19:   **end for**20:   $\bar{g}\leftarrow$ the position of the maximum in $\left\{e_{best,g},\forall g\right\}$.21:   $G_{best}=p_{best,\bar{g}}$.22:   *t* = *t* + 1.23:
**end while**
24:$\theta =G_{best}$.25:Perform user clustereing with the obtained θ based on Algorithm 2.


## 5. Power Allocation for MIMO-NOMA

User clustering and beam-forming are jointly solved in Section 4. However, FTPC is still applied for intra-cluster power allocation, which needs further improvements. In this section, power allocation is optimized based on the optimal user clustering and beam-forming schemes. Without loss of generality, the users in $U_{n}$ are re-ordered in the descending order of effective channel gains. The re-ordered sequence can be denoted by ${\tilde{U}}_{n}=\left\{{\tilde{i}}_{n}\left(1\right),{\tilde{i}}_{n}\left(2\right),\ldots ,{\tilde{i}}_{n}\left(m_{n}\right)\right\}$, where ${\tilde{i}}_{n}\left(l\right)$ ($l\in \{1,2,\ldots ,m_{n}\}$) denotes the index of the *l*-th user in ${\tilde{U}}_{n}$. Moreover, we have ${\left|w_{n}^{H}h_{{\tilde{i}}_{n}\left(1\right)}\right|}^{2}>{\left|w_{n}^{H}h_{{\tilde{i}}_{n}\left(2\right)}\right|}^{2}>\ldots >{\left|w_{n}^{H}h_{{\tilde{i}}_{n}\left(m_{n}\right)}\right|}^{2}$, where $h_{{\tilde{i}}_{n}\left(l\right)}$ denotes the channel vector of user ${\tilde{i}}_{n}\left(l\right)$. The achievable rate of user ${\tilde{i}}_{n}\left(l\right)$ with normalized bandwidth can be represented as:

(22) $$\begin{array}{cc} R_{{\tilde{i}}_{n}\left(l\right)} & =\log(1+\frac{p_{{\tilde{i}}_{n}\left(l\right)}g_{{\tilde{i}}_{n}\left(l\right)}}{1+\sum_{b=1}^{l-1}p_{{\tilde{i}}_{n}\left(b\right)}g_{{\tilde{i}}_{n}\left(l\right)}})\\ \; & =\log\left(1+\sum_{b=1}^{l}p_{{\tilde{i}}_{n}\left(b\right)}g_{{\tilde{i}}_{n}\left(l\right)}\right)-\log\left(1+\sum_{b=1}^{l-1}p_{{\tilde{i}}_{n}\left(b\right)}g_{{\tilde{i}}_{n}\left(l\right)}\right) \end{array}$$

(23) $$g_{{\tilde{i}}_{n}\left(l\right)}=\frac{{\left|w_{n}^{H}h_{{\tilde{i}}_{n}\left(l\right)}\right|}^{2}}{{\left|\omega_{{\tilde{i}}_{n}\left(l\right)}\right|}^{2}}$$

The power allocation problem can be mathematically expressed as below:

(24a) $$\max_{P}:\sum_{n=1}^{S}\sum_{l=1}^{m_{n}}R_{{\tilde{i}}_{n}\left(l\right)}$$

(24b) $$s.t.\frac{p_{{\tilde{i}}_{n}\left(l\right)}g_{{\tilde{i}}_{n}\left(b\right)}}{1+\sum_{j=1}^{l-1}p_{{\tilde{i}}_{n}\left(j\right)}g_{{\tilde{i}}_{n}\left(b\right)}}>\Gamma ,\forall b,l\in \left\{1,2,\ldots ,m_{n}\right\},b<l$$

(24c) $$\sum_{n=1}^{S}\sum_{l=1}^{m_{n}}p_{{\tilde{i}}_{n}\left(l\right)}\leq P_{tot}$$

As mentioned in Section 2, $P=\{p_{k},k=1,2,\ldots ,K\}$ denotes the power allocation scheme. (24b) denotes the SINR constraint for decoding. In each cluster *n*, the signals of user ${\tilde{i}}_{n}\left(l\right)$ should be decoded from the others with higher channel gains. The series SINR constraints can be represented as below:

(25) $$\frac{p_{{\tilde{i}}_{n}\left(l\right)}g_{{\tilde{i}}_{n}\left(l-1\right)}}{1+\sum_{j=1}^{l-1}p_{{\tilde{i}}_{n}\left(j\right)}g_{{\tilde{i}}_{n}\left(l-1\right)}}>\Gamma$$

(26) $$\frac{p_{{\tilde{i}}_{n}\left(l\right)}g_{{\tilde{i}}_{n}\left(l-2\right)}}{1+\sum_{j=1}^{l-1}p_{{\tilde{i}}_{n}\left(j\right)}g_{{\tilde{i}}_{n}\left(l-2\right)}}>\Gamma$$

⋮

(27) $$\frac{p_{{\tilde{i}}_{n}\left(l\right)}g_{{\tilde{i}}_{n}\left(1\right)}}{1+\sum_{j=1}^{l-1}p_{{\tilde{i}}_{n}\left(j\right)}g_{{\tilde{i}}_{n}\left(1\right)}}>\Gamma$$

Since $g_{{\tilde{i}}_{n}\left(1\right)}>g_{{\tilde{i}}_{n}\left(2\right)}>\ldots >g_{{\tilde{i}}_{n}\left(m_{n}\right)}$, (25)–(27) will all hold when (25) holds. The considered problem can be further simplified as:

(28a) $$\max_{P}:\sum_{n=1}^{S}\sum_{l=1}^{m_{n}}R_{{\tilde{i}}_{n}\left(l\right)}$$

(28b) $$s.t.\frac{p_{{\tilde{i}}_{n}\left(l\right)}g_{{\tilde{i}}_{n}\left(l-1\right)}}{1+\sum_{j=1}^{l-1}p_{{\tilde{i}}_{n}\left(j\right)}g_{{\tilde{i}}_{n}\left(l-1\right)}}>\Gamma ,\forall l=2,3,\ldots ,m_{n}$$

(28c) $$\sum_{n=1}^{S}\sum_{l=1}^{m_{n}}p_{{\tilde{i}}_{n}\left(l\right)}\leq P_{tot}$$

The power budget of each cluster can be auto-adjusted based on the channel characteristics of intra-cluster users. To solve (28), we first introduce an auxiliary variable *t* to bound (28a) from below and then optimize (28) by maximizing *t*. The equivalence problem is given by

(29a) $$\max_{t,P}:t$$

(29b) $$s.t.\sum_{n=1}^{S}\sum_{l=1}^{m_{n}}\log\left(1+\sum_{b=1}^{l}p_{{\tilde{i}}_{n}\left(b\right)}g_{{\tilde{i}}_{n}\left(l\right)}\right)-\log\left(1+\sum_{b=1}^{l-1}p_{{\tilde{i}}_{n}\left(b\right)}g_{{\tilde{i}}_{n}\left(l\right)}\right)>t$$

(29c) $$p_{{\tilde{i}}_{n}\left(l\right)}g_{{\tilde{i}}_{n}\left(l-1\right)}-\Gamma \left(1+\sum_{j=1}^{l-1}p_{{\tilde{i}}_{n}\left(j\right)}g_{{\tilde{i}}_{n}\left(l-1\right)}\right)>0,\forall l=2,3,\ldots ,m_{n}$$

(29d) $$\sum_{n=1}^{S}\sum_{l=1}^{m_{n}}p_{{\tilde{i}}_{n}\left(l\right)}\leq P_{tot}$$

However, problem (29) is non-convex since (29b) is a non-convex constraint. To address this issue, we produce a convex relaxation of (29b) based on SCA. Accordingly, (29) is transformed into a convex problem, which can be efficiently solved with a polynomial time. To relax (29b), we first consider a DC function, given by

(30) $$\xi_{n,l}=\log\left(1+\sum_{b=1}^{l}p_{{\tilde{i}}_{n}\left(b\right)}g_{{\tilde{i}}_{n}\left(l\right)}\right)-\log\left(1+\sum_{b=1}^{l-1}p_{{\tilde{i}}_{n}\left(b\right)}g_{{\tilde{i}}_{n}\left(l\right)}\right)$$

The first and second term in (30) are both logarithmic functions, which makes (30) a DC function. Due to the concavity of logarithmic functions, the second term in (30) can be tightly bounded from above with its first-order Taylor expansion, i.e., with any given $\left\{{\bar{p}}_{{\tilde{i}}_{n}\left(1\right)},{\bar{p}}_{{\tilde{i}}_{n}\left(2\right)},\ldots ,{\bar{p}}_{{\tilde{i}}_{n}\left(l-1\right)}\right\}$, we have

(31) $$\begin{array}{cc} \log(1+\sum_{b=1}^{l-1}p_{{\tilde{i}}_{n}\left(b\right)}g_{{\tilde{i}}_{n}\left(l\right)})< & \log(1+\sum_{b=1}^{l-1}{\bar{p}}_{{\tilde{i}}_{n}\left(b\right)}g_{{\tilde{i}}_{n}\left(l\right)})\\ & +\frac{g_{{\tilde{i}}_{n}\left(l\right)}}{1+\sum_{b=1}^{l-1}{\bar{p}}_{{\tilde{i}}_{n}\left(b\right)}g_{{\tilde{i}}_{n}\left(l\right)}}\sum_{b=1}^{l-1}\left(p_{{\tilde{i}}_{n}\left(b\right)}-{\bar{p}}_{{\tilde{i}}_{n}\left(b\right)}\right) \end{array}$$

Substitute (31) into (30), we obtain

(32) $$\begin{array}{cc} \xi_{n,l}\;> & \log\left(1+\sum_{b=1}^{l}p_{{\tilde{i}}_{n}\left(b\right)}g_{{\tilde{i}}_{n}\left(l\right)}\right)-\log\left(1+\sum_{b=1}^{l-1}{\bar{p}}_{{\tilde{i}}_{n}\left(b\right)}g_{{\tilde{i}}_{n}\left(l\right)}\right)\\ & -\frac{g_{{\tilde{i}}_{n}\left(l\right)}}{1+\sum_{b=1}^{l-1}{\bar{p}}_{{\tilde{i}}_{n}\left(b\right)}g_{{\tilde{i}}_{n}\left(l\right)}}\sum_{b=1}^{l-1}\left(p_{{\tilde{i}}_{n}\left(b\right)}-{\bar{p}}_{{\tilde{i}}_{n}\left(b\right)}\right) \end{array}$$

The left-hand side (LHS) of (29b) can be represented as $\sum_{n=1}^{S}\sum_{l=1}^{m_{n}}\xi_{n,l}$. Accordingly, with any given $\left\{{\bar{p}}_{k},k=1,2,\ldots ,K\right\}$, we can derive a lower bound *B* for the LHS of (29b), represented as (33) from the top of next page. Obviously, *B* is convex in P, and (29b) can be further relaxed by restricting *B* to be greater than *t*. The equivalence convex problem is given by (34)–(37).

(33) $$\begin{array}{c} \sum_{n=1}^{S}\sum_{l=1}^{m_{n}}\log\left(1+\sum_{b=1}^{l}p_{{\tilde{i}}_{n}\left(b\right)}g_{{\tilde{i}}_{n}\left(l\right)}\right)-\log\left(1+\sum_{b=1}^{l-1}p_{{\tilde{i}}_{n}\left(b\right)}g_{{\tilde{i}}_{n}\left(l\right)}\right)>\underset{B}{\underset{︸}{\sum_{n=1}^{S}\sum_{l=1}^{m_{n}}\left(\log\left(1+\sum_{b=1}^{l}p_{{\tilde{i}}_{n}\left(b\right)}g_{{\tilde{i}}_{n}\left(l\right)}\right)-\log\left(1+\sum_{b=1}^{l-1}{\bar{p}}_{{\tilde{i}}_{n}\left(b\right)}g_{{\tilde{i}}_{n}\left(l\right)}\right)-\frac{g_{{\tilde{i}}_{n}\left(l\right)}}{1+\sum_{b=1}^{l-1}{\bar{p}}_{{\tilde{i}}_{n}\left(b\right)}g_{{\tilde{i}}_{n}\left(l\right)}}\sum_{b=1}^{l-1}\left(p_{{\tilde{i}}_{n}\left(b\right)}-{\bar{p}}_{{\tilde{i}}_{n}\left(b\right)}\right)\right)}} \end{array}$$

(34) $$\max_{t,P}:t$$

(35) s.t.B>t

(36) $$p_{{\tilde{i}}_{n}\left(l\right)}g_{{\tilde{i}}_{n}\left(l-1\right)}-\Gamma \left(1+\sum_{j=1}^{l-1}p_{{\tilde{i}}_{n}\left(j\right)}g_{{\tilde{i}}_{n}\left(l-1\right)}\right)>0,\forall l=2,3,\ldots ,m_{n}$$

(37) $$P_{tot}-\sum_{n=1}^{S}\sum_{l=1}^{m_{n}}p_{{\tilde{i}}_{n}\left(l\right)}\geq 0$$

According to the principles of SCA, the solution of problem (29) should be obtained through multiple iterations. In each iteration, we produce an equivalence problem of (29) as (34)–(37), which is further solved by some effective optimization tools. Specifically, in the *i*-th iteration, $\left\{{\bar{p}}_{k},k=1,2,\ldots ,K\right\}$ should be set as the solution obtained in the previous iteration. More details are as described in Algorithm 4.**Algorithm 4** Power Allocation Scheme for MIMO-NOMA1:${\bar{p}}_{{\tilde{i}}_{n}\left(l\right)}=\frac{P_{tot}}{S}\frac{g_{{\tilde{i}}_{n}\left(l\right)}^{-\gamma }}{\sum_{j=1}^{m_{n}}g_{{\tilde{i}}_{n}\left(j\right)}^{-\gamma }},\forall n,l$.2:Initialize η.3:$r_{0}\leftarrow \sum_{n=1}^{S}\sum_{l=1}^{m_{n}}\log(1+\frac{{\bar{p}}_{{\tilde{i}}_{n}\left(l\right)}g_{{\tilde{i}}_{n}\left(l\right)}}{1+\sum_{b=1}^{l-1}{\bar{p}}_{{\tilde{i}}_{n}\left(b\right)}g_{{\tilde{i}}_{n}\left(l\right)}})$.4:*i* = 0.5:**repeat**6:   *i* = *i* + 1.7:   Produce an equivalence problem of (29) as (34)–(37) based on the Taylor expansion at $\left\{{\bar{p}}_{k},\forall k\right\}$.8:   Solve the obtained convex problem to get $\left\{{\tilde{p}}_{k},\forall k\right\}$.9:   $r_{i}\leftarrow \sum_{n=1}^{S}\sum_{l=1}^{m_{n}}\log(1+\frac{{\tilde{p}}_{{\tilde{i}}_{n}\left(l\right)}g_{{\tilde{i}}_{n}\left(l\right)}}{1+\sum_{b=1}^{l-1}{\tilde{p}}_{{\tilde{i}}_{n}\left(b\right)}g_{{\tilde{i}}_{n}\left(l\right)}})$.10:   ${\bar{p}}_{k}\leftarrow {\tilde{p}}_{k},\forall k$.11:**until**$\left|\frac{r_{i}-r_{i-1}}{r_{i-1}}\right|<\eta$12:$p_{k}={\bar{p}}_{k},\forall k$.

## 6. Simulations Results

In this section, the performance of the proposed scheme is evaluated by multiple simulations. The distance from users to the BS is uniformly distributed in the range of 0 to 500 m. The channel vector of each user is assumed to be the product of large-scale path loss and Rayleigh fading. We also evaluate the performances of two existing schemes to illustrate the significance of the proposed scheme [9,21]. Some key parameters are as summarized in Table 1. The effects of multiple factors will be discussed in more details.

**Figure 1:**
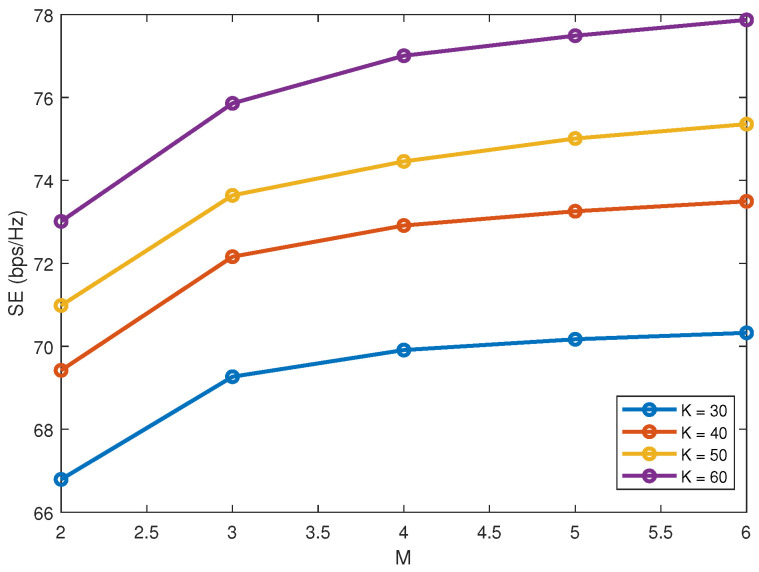
Spectrum efficiency (SE) versus *M*.

Figure 1 plots SE versus *M* with γ = 0.2 and *S* = 2. As shown in Figure 1, SE increases with *M* since a larger *M* allows a cluster to serve more users. However, such effect gets saturated as *M* increases to a certain degree, subject to the total power budget.

**Figure 2:**
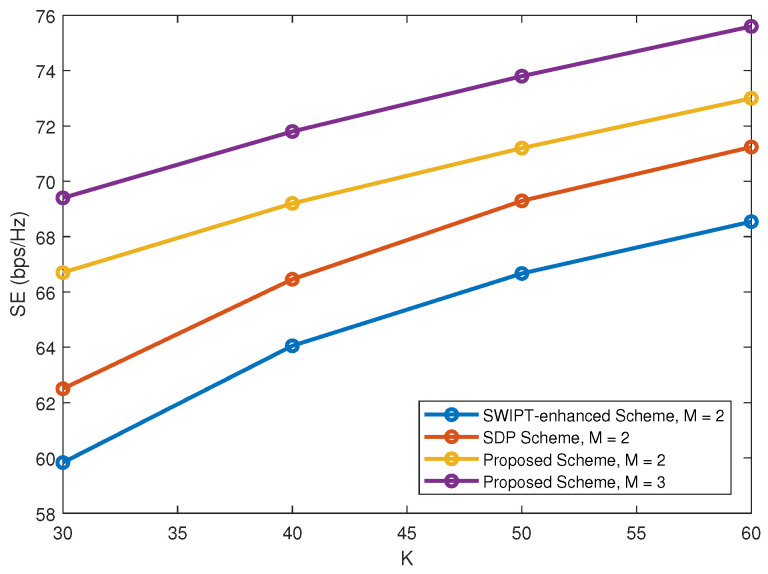
SE versus *K*.

The effect of user diversity is also considered. Figure 2 plots SE versus *K* with γ = 0.2 and *S* = 2. From Figure 2, one can observe that user diversity plays a key role in increasing SE. Moreover, the proposed scheme outperforms the two existing schemes in terms of SE.

**Figure 3:**
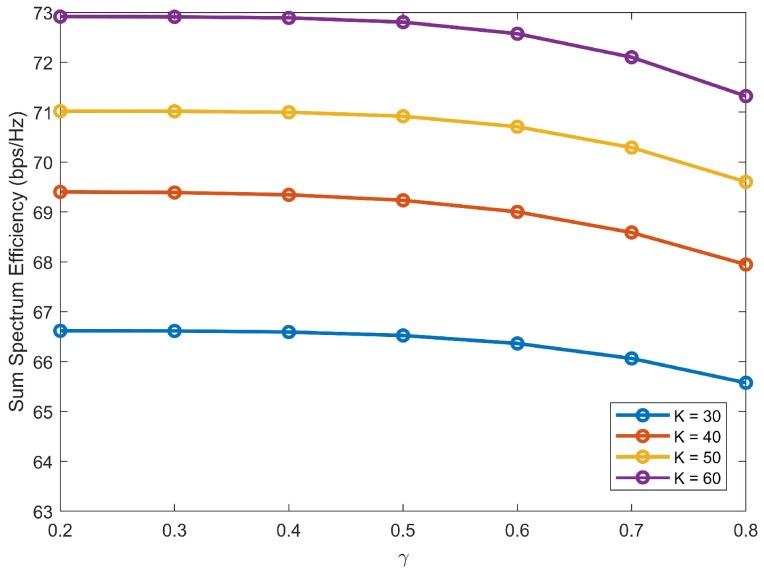
SE versus γ.

Next, the impact of the decay parameter γ is further considered. In Algorithm 1, γ determines the correlation between users’ channel gains and transmitting power. As γ increases, weak users have made notable gains at the expense of strong users. However, the achievable SE mainly depends on strong users due to their high channel gains. Figure 3 plots SE versus γ with *S* = 2 and *M* = 2. From Figure 3, SE decreases with the increase of γ due to the performances degradation of strong users.

**Figure 4:**
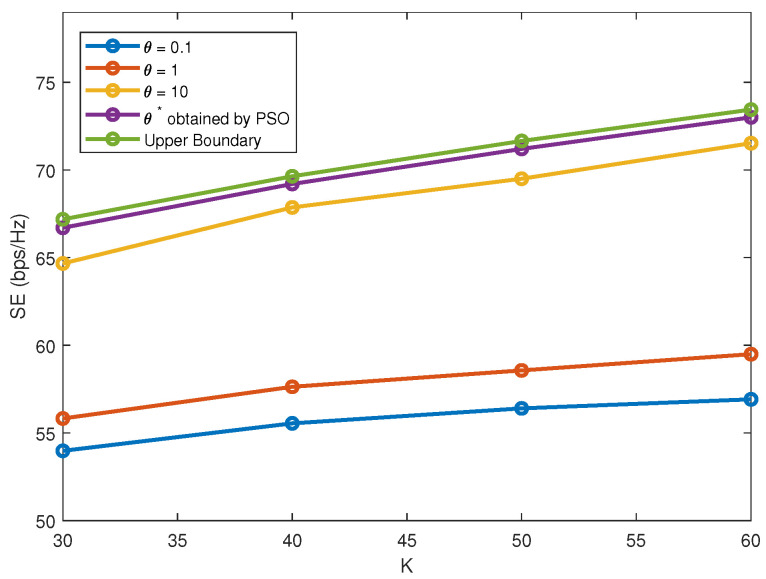
SE versus *K* under different θ.

In Algorithm 2, channel differences and correlations are both involved in the design of user clustering. A relative weight θ is introduced for the two aspects, which is obtained based on group hunting strategy. As discussed before, θ is of great significance in steering the ultimate clustering scheme. With *M* = 2, γ = 0.2 and *S* = 2, Figure 4 plots SE versus *K* under different θ. When θ = 0.1, the achievable SE is relatively small because of neglect of channel difference characteristics. As θ increases, we can achieve a better balance between the two contributing factors and SE will increase accordingly. However, when θ increases to a certain degree, e.g., θ≥10, the achievable SE will further decrease for overlooking channel correlations characteristics. Moreover, the performance upper bound is also considered. Exhaustive user search can be done to find the optimal clustering scheme. Based on Algorithms 1 and 4, beam-forming and power allocation can be done for each possible clustering scheme to compare their performance. From Figure 4, the performance of the proposed scheme can approach to the upper bound due to the optimization strategy of PSO.

**Figure 5:**
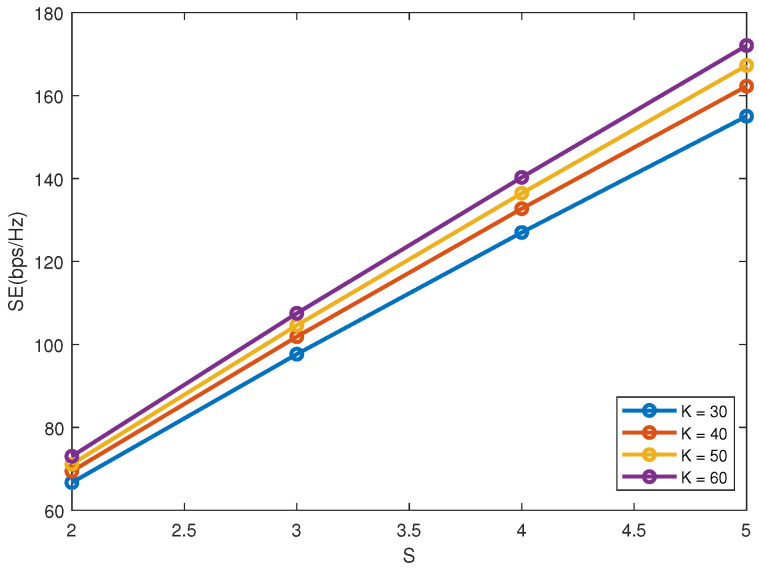
SE versus *S*.

Figure 5 plots SE versus *S* with *M* = 2 and γ = 0.2. The number of users that can be served simultaneously will increase with the increase of *S*. Moreover, when N≥S−1, there is no interference among different clusters. As shown in Figure 5, SE has a nearly linear increase with *S* due to the orthogonality among different clusters.

**Figure 6:**
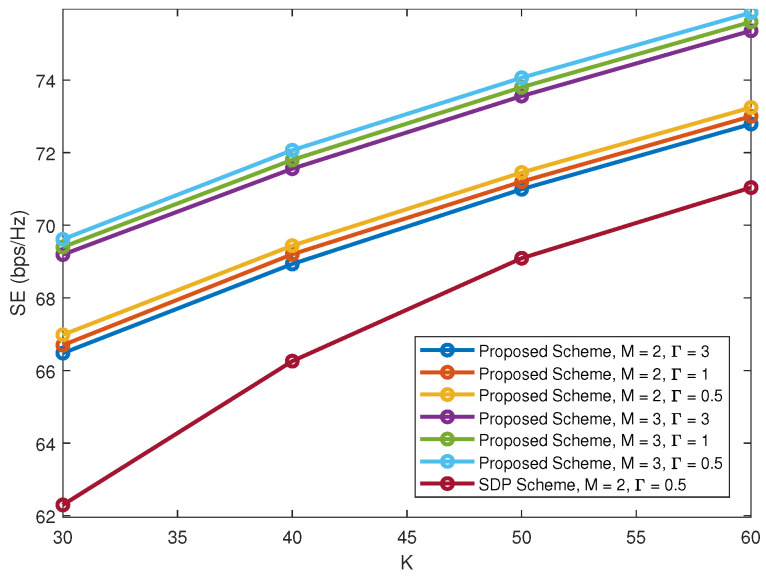
SE versus *K* under different Γ.

The effect of Γ is also considered. In the SIC-based decoding process, the received SINR at users will increase with the increase of Γ, which not only improves system reliability, but also enables more power budget for edge users. Accordingly, as Γ increases, there is a corresponding increase in edge users’ data rates. With γ = 0.2, Figure 6 plots SE versus *K* under different Γ. From Figure 6, the achievable SE is almost unaffected by Γ, i.e., as Γ increases, we sacrifice strong users’ data rates in exchange for weak users’ rates to ensure all multiplexed users can achieve satisfactory performances. However, the existing schemes fail to obtain a feasible solution when Γ is greater than 0.5. By contrast, the proposed scheme is more robust which can obtain a feasible solution with a larger Γ. One explanation for this is that, the proposed scheme solves beam-forming and power allocation separately, which predigests the scope of the considered problem and helps to achieve a better performance with strong robustness.

**Figure 7:**
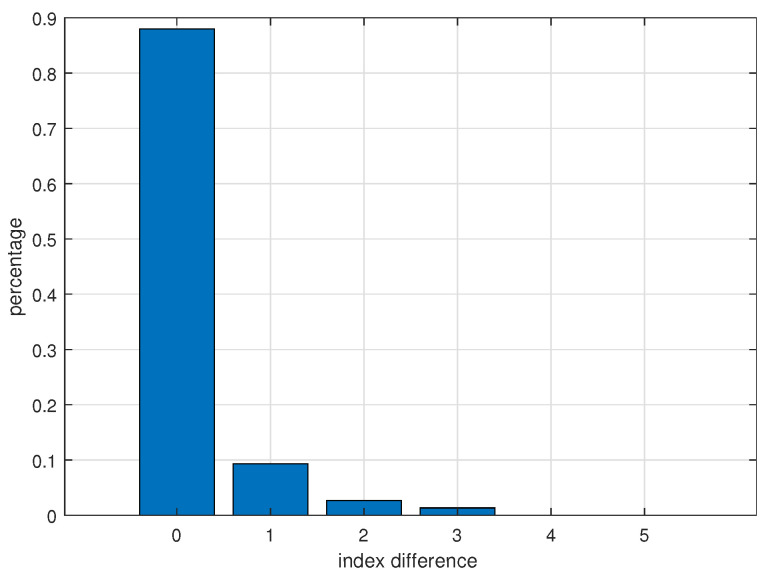
Position difference distribution.

The effect of beam-forming on the optimal decoding order is also investigated. We generate 1000 instances with γ = 0.2, *K* = 60, *S* = 5 and *M* = 6. The proposed scheme is applied for the realization of each instance. In each cluster, the intra-cluster users are sorted in the descending order of channel gains and normalized channel gains, respectively. The positions of each user in the two sorted sequences are recorded, and their difference can be utilized to describe how often beam-forming changes the optimal SIC order. Figure 7 plots the distribution of the position differences. From Figure 7, the decoding order generally remains unchanged. However, there are circumstances where the optimal SIC order is slightly adjusted.

### Complexity Analysis

With any given relative weight, the corresponding clustering scheme can be obtained by Algorithm 2. For each user *k*, Algorithm 2 measures *k*’ s preference for different clusters.

User *k* will be first assigned to its favorite cluster $n_{k}$. After assigning user k to cluster $n_{k}$, two cases can occur:

- Case 1: The number of the users in cluster $n_{k}$ is no greater than M;
- Case 2: The number of the users in cluster $n_{k}$ is greater than M.

In case 1, user *k* can be directly assigned to cluster $n_{k}$. In case 2, cluster $n_{k}$ should reject a user to meet the size constraint. Algorithm 2 produces (*M* + 1) possible user set for cluster $n_{k}$. Based on Algorithm 1, beam-forming can be done for each possible user set to compare their performance, and the rejected user is obtained accordingly. If the rejected user is user *k*, *k* will be further assigned to its second-favorite cluster. The above process will be repeatedly executed until either user *k* is successfully assigned to a single cluster or all the clusters are processed.

Accordingly, Algorithm 2 consists of two parts: part 1 measures each user’s preference for different clusters; part 2 helps each user select a suitable cluster to join. The complexity of part 1 is O(SK), and the complexity of part 2 is O(SK). The complexity of Algorithm 2 is O(SK).

The optimal relative weight can be obtained by Algorithm 3 through *D* iterations. In each iteration, Algorithm 3 produces *G* possible relative weights, each corresponding to a possible clustering scheme. Based on Algorithm 1, beam-forming can be done for each possible clustering scheme to compare their performance. The complexity of Algorithm 3 is O(SK).

## 7. Conclusions

In this passage, we consider the multi-dimensional resource allocation problem for MIMO-NOMA, which consists of power allocation, user clustering and beam-forming, respectively. A three-step resource allocation framework is proposed to solve the considered problem: step 1 solves the beam-forming problem for a given user cluster; step 2 obtains the optimal clustering scheme based on the proposed beam-forming algorithm; step 3 further optimizes power allocation based on the optimal user clustering and beam-forming schemes. Simulation results show that the proposed scheme can effectively increase the received SINR at users. Additionally, the performance of the proposed scheme can approach the performance upper bound in terms of SE.

## Figures and Tables

**Table 1:** Simulation Parameters.

Parameter	Value
number of antennas (*N*)	2
noise power spectral density ($N_{0}$)	−169 dBm
system bandwidth (*B*)	360 kHz
large-scale path loss model	free-space path loss model
*T*	20
η	$10^{-3}$
*G*	30
$P_{tot}$	30 dBm
*D*	100

## Appendix A

The normalized channel gain of user *l* is given by

(A1) $$\begin{array}{cc} g_{l} & =\frac{1}{{\left|{\bar{\omega }}_{l}\right|}^{2}}{\left|h_{l}^{H}w_{n}\right|}^{2}=\frac{1}{{\left|{\bar{\omega }}_{l}\right|}^{2}}{\left|\frac{1}{\sqrt{N}}\sum_{c=1}^{N}\kappa_{l,c}e^{j(\phi_{c}-\varphi_{l,c})}\right|}^{2} \end{array}$$

(A2) $$\begin{array}{cc} \; & =\frac{1}{{\left|{\bar{\omega }}_{l}\right|}^{2}}{\left|\frac{1}{\sqrt{N}}\sum_{c=1}^{N}\kappa_{l,c}\cos\left(\phi_{c}-\varphi_{l,c}\right)+j\frac{1}{\sqrt{N}}\sum_{c=1}^{N}\kappa_{l,c}\sin\left(\phi_{c}-\varphi_{l,c}\right)\right|}^{2} \end{array}$$

(A3) $$\begin{array}{cc} \; & =\frac{1}{{\left|{\bar{\omega }}_{l}\right|}^{2}N}{\left(\sum_{c=1}^{N}\kappa_{l,c}\cos\left(\phi_{c}-\varphi_{l,c}\right)\right)}^{2}+\frac{1}{{\left|{\bar{\omega }}_{l}\right|}^{2}N}{\left(\sum_{c=1}^{N}\kappa_{l,c}\sin\left(\phi_{c}-\varphi_{l,c}\right)\right)}^{2} \end{array}$$

(A4) $$\begin{array}{cc} \; & =\frac{1}{{\left|{\bar{\omega }}_{l}\right|}^{2}N}\sum_{c=1}^{N}\kappa_{l,c}^{2}\cos^{2}\left(\phi_{c}-\varphi_{l,c}\right)+\frac{1}{{\left|{\bar{\omega }}_{l}\right|}^{2}N}\sum_{c=1}^{N}\kappa_{l,c}^{2}\sin^{2}\left(\phi_{c}-\varphi_{l,c}\right) \end{array}$$

(A5) $$\begin{array}{cc} & +\frac{2}{{\left|{\bar{\omega }}_{l}\right|}^{2}N}\sum_{c=1}^{N}\sum_{d=c+1}^{N}\kappa_{l,c}\kappa_{l,d}\cos\left(\phi_{c}-\varphi_{l,c}\right)\cos\left(\phi_{d}-\varphi_{l,d}\right) \end{array}$$

(A6) $$\begin{array}{cc} & +\frac{2}{{\left|{\bar{\omega }}_{l}\right|}^{2}N}\sum_{c=1}^{N}\sum_{d=c+1}^{N}\kappa_{l,c}\kappa_{l,d}\sin\left(\phi_{c}-\varphi_{l,c}\right)\sin\left(\phi_{d}-\varphi_{l,d}\right) \end{array}$$

(A7) $$\begin{array}{cc} \; & =\frac{1}{{\left|{\bar{\omega }}_{l}\right|}^{2}N}\sum_{c=1}^{N}\kappa_{l,c}^{2}+\frac{2}{{\left|{\bar{\omega }}_{l}\right|}^{2}N}\sum_{c=1}^{N}\sum_{d=c+1}^{N}\kappa_{l,c}\kappa_{l,d}\cos(\phi_{c}-\varphi_{l,c}-\left(\phi_{d}-\varphi_{l,d}\right)) \end{array}$$

(A8) $$\begin{array}{cc} \; & =\frac{1}{{\left|{\bar{\omega }}_{l}\right|}^{2}N}{∥h_{l}∥}_{2}^{2}+\frac{2}{{\left|{\bar{\omega }}_{l}\right|}^{2}N}\sum_{c=1}^{N}\sum_{d=c+1}^{N}\kappa_{l,c}\kappa_{l,d}\cos(\phi_{c}-\varphi_{l,c}-\left(\phi_{d}-\varphi_{l,d}\right)) \end{array}$$

where $\kappa_{l,c}$ and $\varphi_{l,c}$ denote the amplitude and phase of the *c*-th element in $h_{l}$, respectively.
